# 

**Published:** 1876-06

**Authors:** 


### Books and Pamphlets Received.

The Medical and Surgical History of the War of the Rebellion. Part II, Vol. II. Surgical History. Prepared under the direction of Joseph K. Barnes, Surgeon General U. S. Army, Washington: Government Printing Office, 1876.

Statistics, Medical and Authropological, of the Provost-Marshal General's Bureau. Derived from the Records of the examination for Military Service in the armies of the United States, during the late War of the Rebellion, of over a million recruits, drafted men, subsitutes and enrolled men. Compiled under direction of the Secretary of War, by J. H. Baxter, A. M., M. D., Col. and Chief Medical Purveyor, U. S. Army. In two volumes. Washington: Government Printing Office.

Micro-Photographs in Histology, Normal and Pathological. By Carl Seiler, M. D., in conjunction with J. Gibbons Hunt, M. D., and Joseph G. Richardson, M. D. Vol. I, Nos. 1 and 2. Philadelphia: J. H. Coates & Co.

Spiritualism and Allied Causes and Conditions of Nervous Derangement. By William A. Hammond, M. D. Illustrated. New York: G. P. Putnam's Sons, 1876. Buffalo: Peter Paul & Bro.

Cyclopædia of the Practice of Medicine. Edited by Dr. H. Von Ziemssen. Vol. XI, Diseases of the Peripheral Cerebro-Spinal Nerves. By Professor Wilhelm Heinrich Erb, translated by Henry Power, of London, England. A. H. Buck, M. D., American Editor. New York: Wm. Wood & Co., 1876.

Memoires sur la Galvano Caustique Thermique. Par Le Docteur A. Amussat, Filés Avec 44 Figures intercalles dans le Texte. Paris: Librairie de Germer Bailliére, 1876.

Des Sondes a Demeure et du Conducteur en Baleine. Par Le Docteur A. Amussat. Paris: Librairie de Germer Balliére, 1876.

### Contents of June Number.

### ORIGINAL.

THE BUFFALO

### Medical and Surgical Journal

PUBLISHED MONTHLY,

Containing Original Articles, Reports of Medical Societies and Hospitals, Editorial, Reviews, Correspondence, Army News, etc., etc ; including the usual variety of Medical Periodical Publications. Specimen copies sent on application.

Address Buffalo Medical & Surgical Journal, Buffalo, N. Y.

TERMS OF SUBSCRIPTION, $3.00 PER YEAR, IN ADVANCE.

